# Ambient data-driven SSO online monitoring of type-3 wind turbine generator integrated power systems based on MMPF-KF method

**DOI:** 10.1038/s41598-023-42729-3

**Published:** 2023-09-22

**Authors:** Xi Chen, Xi Wu, Jinyu Zhou, Qingfeng Li, Chenyu Wu, Qiang Li, Bixing Ren, Ke Xu

**Affiliations:** 1 School of Electrical Engineering, Southeast University, Nanjing, 210096 China; 2 The State Grid Jiangsu Electric Power Co., Ltd. Research Institute, Nanjing, 211103 China

**Keywords:** Electrical and electronic engineering, Energy infrastructure

## Abstract

Series compensation grids connected with type-3 wind turbine generator (WTG)-based wind farms have suffered numerous subsynchronous oscillation (SSO) events worldwide. For early alerting of SSO and effective development of protection and control strategies, it is critical to monitor and identify SSO accurately and quickly. Ambient data is continuously available, which is useful for online monitoring. This paper proposes an ambient data-driven SSO online monitoring method based on the Kalman filter (KF) combined with the multi-model partitioning filter (MMPF). The KF is utilized to fit the measured ambient data with an auto regressive (AR) model. Then, the damping factor (or damping ratio) and frequency in the SSO mode can be acquired by solving the roots of the characteristic polynomial corresponding to the AR model. Moreover, the MMPF is an effective model order selection method applied to the KF for better identification. The performance of the MMPF-KF method is demonstrated by simulations and real-time experiments. The results of case studies validate the effectiveness of the proposed method under various conditions.

## Introduction

Recently, the proportion of power generation from renewable sources has surged in today’s power systems, leading to the increasingly frequent occurrence of sub-synchronous oscillations (SSOs). Type-3 wind turbine generator (WTG) is one of the most extensively applied renewable power generation equipment, and wind farms composed of such types of WTGs have been subjected to multiple SSO events worldwide, i.e., in north China^1^, southwest Minnesota^2^, and Texas^3^. SSO poses a significant threat, not only to the safety of the electrical equipment but also to the stability of the power system, which can cause severe damage to WTGs^4^, lead to numerous WTGs tripping^5^, and even cause a major power outage^6^. Accurate online monitoring of SSO is of great practical significance since it is crucial for early alerting of SSO and developing protection and control strategies effectively.

Existing methods to monitor SSO can be briefly divided into two groups: the ringdown methods and the ambient methods. Ringdown data indicate the responses of the system to a severe disturbance, such as the tripping of power lines, and they contain strong SSOs. Typical ringdown data-based methods are Prony^7^, discrete Fourier transform (DFT)^8^, and their improved approaches, which have achieved very good performance. However, the infrequent presence of severe disturbances renders the continuous identification of SSO difficult^9,10^. Ambient data are collected from normal operating power systems with small random load or renewable generation fluctuations, which are generally modeled as Gaussian white noise^11^. They are prevalent in measurement signals and include oscillation mode information about a system. Since ambient data is continually accessible, analyzing it is valuable for online monitoring and identification^12^. Unfortunately, since SSOs contained in the ambient data are very weak, the typical ringdown data-based methods above are difficult to apply to the ambient data-based identification of SSOs.

Multiple approaches have been developed for ambient measurements to identify the low frequency oscillation (LFO), such as the wavelet transform (WT)^11^, stochastic subspace identification (SSI)^13^, and empirical mode decomposition (EMD)^14^. However, very little literature has focused on SSO online monitoring based on ambient data. Since the mechanism of SSO is quite different from that of LFO and the frequency of SSO is much higher than that of LFO, whether the existing identification method for LFO is applicable to SSO needs further study. Ref.^15^ utilized the frequency domain decomposition (FDD) method for monitoring SSOs using ambient data for the first time. However, FDD, as a kind of frequency domain algorithm, requires a long data window for accurate estimation of power spectrum density^16^, which significantly affects the detection speed, while a much shorter window length corresponds to inaccurate estimation results.

The Kalman filter (KF) approach, which has been widely used for online synchrophasor estimation^17,18^, can provide accurate identification results in a recursive manner. It does not require an observation window and converges very fast, allowing real-time optimal estimates of an incoming signal. Thus, the KF fits the ambient data-based SSO online monitoring well.

To overcome the limitations of existing ambient data-based SSO monitoring methods, this paper exploits the above advantages of the KF and proposes a KF-based method combined with a multi-model partitioning filter (MMPF), referred to as MMPF-KF, for ambient data-based identification of SSO modes in type-3 WTG integrated power systems. The contributions are listed below:

1. The KF-based algorithm is employed to recursively estimate the frequency and damping ratio of the SSO from the ambient data. The KF-based algorithm achieves high-speed detection while simultaneously ensuring satisfactory estimation accuracy.
2. The MMPF, as an effective model order selection method, is introduced to determine the correct order of the auto-regressive (AR) model used in the KF-based SSO monitoring algorithm for better identifications.
3. The performance of the proposed ambient data-driven SSO online monitoring method is verified in a classical second-order system and a test system of type-3 WTG-based wind farms in north China. A comparison with the existing ambient data-driven SSO monitoring method highlights the superior performance of the proposed method in terms of both calculation time and accuracy. The real-time experiments show the feasibility of the proposed method in practical applications.

This paper is organized as follows: In Section “SSO triggered by the type-3 WTG-based grid with series compensation”, the mechanism of SSO triggered by the type-3 WTG-based power grid is introduced to give the reader an overall understanding of the research object of this paper. Then, the ambient data-driven SSO mode identification method using MMPF-KF is proposed in Section “Ambient data-driven SSO online monitoring method using MMPF-KF”. In Section “Case studies”, the implementation and evaluation of the proposed method are presented. Finally, the conclusions are given in Section “Conclusions”.

## SSO triggered by the type-3 WTG-based grid with series compensation

**Figure 1:**
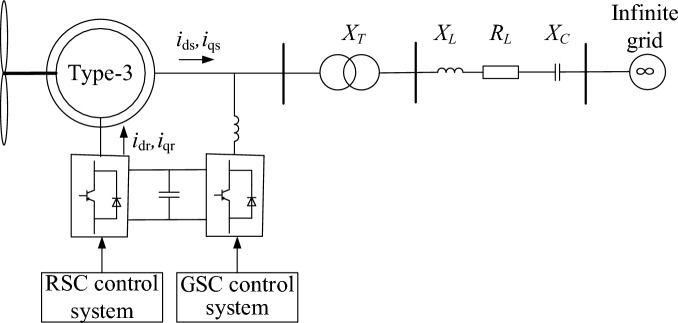
Schematic diagram of the type-3 WTG integrated power system.

The structure of the type-3 WTG aggregated wind system is shown in Fig. 1. As wind farms are generally located far from the point of common coupling (PCC)^19^, the transmission line is typically series-capacitor-compensated to enhance the capability of transferring power. However, the configuration of series compensation may reduce the stability of the type-3 WTG-connected power system, leading to a severe SSO^20^. Extensive research has suggested that interplays between the control systems of type-3 WTGs and the series-compensated networks^21^. The rotor side converter (RSC) of the type-3 WTG can exhibit negative resistance to the external network, and SSO would be triggered in the case of the negative resistance overcoming the positive one of the compensated networks^22^. The stability level is affected by several factors, i.e., wind speed, parameters of the RSC controller, compensation level, and length of transmission line^23^. In general, the parameters of the RSC controller, the compensation level, and the length of the transmission line of a system are fixed, while the wind speed can vary with time, resulting in changes in the system stability level. Since the system stability level can be reflected in the SSO mode, continuous SSO mode identification based on ambient data is helpful for early alerting of SSO risks due to changes in operating conditions.

## Ambient data-driven SSO online monitoring method using MMPF-KF

### AR model and principle of the ambient data-based SSO mode identification

An AR model of order *p*, which can be referred to as AR(*p*), is given by (1)^24^:

1 $$y(k) = \sum\limits_{i = 1}^{p} {a_{i} (k)y(k - i)} + v(k)$$

where *y*(*k*) is the measured signal from the measurement device of the power system at time step *k*. In this paper, the measured signal is selected as the output active power of the type-3 WTG based wind farm, which can be easily obtained from broadband measurement devices. *a*_1_(*k*), *a*_2_(*k*), …, *a*_*p*_(*k*) are the time-varying coefficients of the AR model. *v*(*k*) is the white noise.

The characteristic polynomial of AR model (1) is presented as follows:

2 $$z^{p} - a_{1} (k)z^{p - 1} - \cdots - a_{p - 1} (k)z - a_{p} (k) = 0$$

The poles of the above polynomial *z*_*i*_ are identical to the eigenvalues of the power system in the discrete time domain. Then, the eigenvalues in the continuous time domain are calculated using the equation as follows:

3 $$s_{i} = f_{s} \ln z_{i} = \alpha_{i} + j\omega_{i}$$

where *f*_*s*_ is the sampling rate of the measured signal described using AR(*p*). *i* = 1, …, *n*, *n* indicates the total number of oscillation modes present in the system. The real part of eigenvalue *s*_*i*_, denoted by *α*_*i*_, stands for the damping factor of the *i*th oscillation mode, and the imaginary part of *s*_*i*_, denoted by *ω*_*i*_, stands for the angular frequency of the *i*th oscillation mode.

As stated in (3), the frequency and damping information of oscillation modes that characterize the dynamics of the system can be derived from the eigenvalues in the continuous time domain. As long as parameters of AR(*p*) are estimated based on ambient data, it is possible to obtain the eigenvalues of the type-3 WTG integrated power system by solving (2) and (3), and SSO modes of the system can be identified eventually.

### MMPF-KF-based SSO mode identification algorithm

The KF is utilized to estimate the parameters of the AR model of the type-3 WTG-based wind system in this paper. Before parameter estimation, the model order *p* should be determined first. It is important for the SSO identification since an order being too low will result in the missing of true modes, and an order being too high will lead to the presence of fake modes on the contrary^25^. An effective model order selection method, namely MMPF^26^, is introduced, combining with the KF for a better result in SSO identification. The details of the MMPF-KF are as follows.

The AR model (1) can be represented in the standard state-space form as follows:

4 $${\mathbf{x}}_{p} (k) = {\mathbf{x}}_{p} (k - 1)$$

5 $$y(k) = {\mathbf{H}}_{p} (k){\mathbf{x}}_{p} (k) + v(k)$$

where Eq. (4) is called the process equation, and **x**_*p*_(*k*) is the state vector, with **x**_*p*_(*k*) = [*a*_1_(*k*) *a*_2_(*k*) … *a*_*p*_(*k*)]^T^. Equation (5) is called the measurement equation, and **H**_*p*_(*k*) is the observation matrix, with **H**_*p*_(*k*) = [*y*(*k* − 1) *y*(*k* − 2) … *y*(*k* − *p*)].

The estimation of parameters *a*_1_(*k*), *a*_2_(*k*), …, *a*_*p*_(*k*) using KF includes the following steps^27^.

1. Initialization:
  The parameters are initialized as:
  6 $$\left\{ \begin{aligned} {\hat{\mathbf{x}}}_{p} (1) & = [\underbrace {{\begin{array}{*{20}c} 0 & 0 & \ldots & 0 \\ \end{array} }}_{p}]^{{\text{T}}} \\ {\mathbf{P}}_{p} (1) & = n{\mathbf{I}}_{p \times p} \\ \end{aligned} \right.$$
  where **P**_*p*_(1) is the initial value of **P**_*p*_(*k*), which stands for the covariance matrix of the state vector estimation error. **I**_*p*×*p*_ is a *p* × *p* identity matrix, with *n* being a relatively large integer.
2. Forecasting:
  The state vector and its error covariance matrix forecasted at time step *k* are calculated from the previously estimated state vector ${\hat{\mathbf{x}}}_{p} (k - 1)$ and its error covariance matrix **P**_*p*_(*k* − 1) by the following equations:
  7 $${\hat{\mathbf{x}}}_{p} (k\left| {k - 1)} \right. = {\hat{\mathbf{x}}}_{p} (k - 1)$$
  8 $${\mathbf{P}}_{p} (k\left| {k - 1)} \right. = {\mathbf{P}}_{p} (k - 1)$$
  where (*k*|*k* − 1) denotes the forecast at time step *k* based on measurements at time step *k* − 1.
3. Computing Kalman Gain:
  The Kalman gain **K**_*p*_(*k*) is calculated by
  9 $${\mathbf{K}}_{p} (k) = \frac{{{\mathbf{P}}_{p} (k\left| {k - 1)} \right.\mathbf{H}_{p}^{{\text{T}}} (k)}}{{\mathbf{H}_{p} (k)\mathbf{P}_{p} (k\left| {k - 1)} \right.\mathbf{H}_{p}^{{\text{T}}} (k) + R(k)}}$$
  where *R*(*k*) is the covariance of measurement noise *v*(*k*).
4. Rectification
  Refine the obtained forecast of the state vector ${\hat{\mathbf{x}}}_{p} (k\left| {k - 1} \right.)$ with the new incoming measured data *y*(*k*) and the Kalman gain **K**_*p*_(*k*)
  10 $${\hat{\mathbf{x}}}_{p} (k) = {\hat{\mathbf{x}}}_{p} (k\left| {k - 1} \right.) + {\mathbf{K}}_{p} (k)[y(k) - {\mathbf{H}}_{p} (k){\hat{\mathbf{x}}}_{p} (k\left| {k - 1} \right.)]$$

The error covariance matrix of the optimal state estimation is updated as

11 $${\mathbf{P}}_{p} (k) = {\mathbf{P}}_{p} (k\left| {k - 1} \right.) - {\mathbf{K}}_{p} (k){\mathbf{H}}_{p} (k){\mathbf{P}}_{p} (k\left| {k - 1} \right.)$$

Then repeat step 2.

Based on the above KF method, the unknown model order *p* is estimated by MMPF as follows.

It is assumed that the model order *p* is contained within a known finite range that extends from 1 to *M*. Then a bank of KFs is applied to the AR models of each model order. The optimal estimation of **x**_*p*_(*k*) as determined by the minimum mean-square error (MMSE) can be obtained by

12 $${\hat{\mathbf{x}}}_{p} (k) = \sum\limits_{j = 1}^{M} {{\hat{\mathbf{x}}}_{j} (k)} p_{j} (k)$$

where ${\hat{\mathbf{x}}}_{j} (k)$ is the estimated parameter vector of the *j*-order AR model at time step *k* by KF using (10). *p*_*j*_(*k*) is the probability of selecting the AR model of order *j* as the correct one. Generally, prior knowledge of the probability is absent, and the initial value *p*_*j*_(1) can be set to 1/*M*.

The probability for the AR model of order *j* at time step *k* is calculated in a recursive manner by:

13 $$p_{j} (k) = \frac{{L_{j} (k)}}{{\sum\nolimits_{j = 1}^{M} {L_{j} (k)p_{j} (k - 1)} }}p_{j} (k - 1)$$

where

14 $$L_{j} (k) = \left| {Q_{j} (k)} \right|^{ - 1/2} \times \exp \left[ { - \frac{1}{2}\varepsilon_{j}^{2} (k) \times Q_{j}^{ - 1} (k)} \right]$$

15 $$\varepsilon_{j} (k) = y(k) - {\mathbf{H}}_{j} (k){\hat{\mathbf{x}}}_{j} (k|k - 1)$$

16 $$Q_{j} (k) = {\mathbf{H}}_{j} (k){\mathbf{P}}_{j} (k){\mathbf{H}}_{j}^{{\text{T}}} (k) + R(k)$$

Note that ${\hat{\mathbf{x}}}_{j} (k|k - 1)$ of Eq. (15) can be calculated by (7) and **P**_*j*_(*k*) of Eq. (16) can be calculated by (11). At each time step, the model with the highest probability is the one that is chosen as the correct one by the MMPF. The maximum probability usually approaches 1, whereas the other probabilities typically approach 0. It is worth mentioning that all KFs required in the MMPF can be run in parallel, which is a key characteristic of the MMPF; thus, the computational time of identifying SSO modes will not be increased.

Once the parameter vector and the model order are estimated, they can be substituted into (2) for eigenvalue calculation. Then, the eigenvalues can be transformed from the discrete domain to the continuous domain by (3). The oscillation frequency of the *i*th mode can be extracted from the imaginary part of the corresponding eigenvalue by:

17 $$f_{SSOi} (k) = {\text{imag[}}s_{i} (k)]/2\pi$$

with a damping factor extracted from the real part of the eigenvalue:

18 $$\alpha_{SSOi} (k) = {\text{re[}}s_{i} (k)]$$

In the eigenvalue analysis, a negative damping factor indicates a stable mode, whereas a positive damping factor indicates that the mode is unstable. In this paper, the mode with the highest damping factor is selected as the dominant mode.

**Figure 2:**
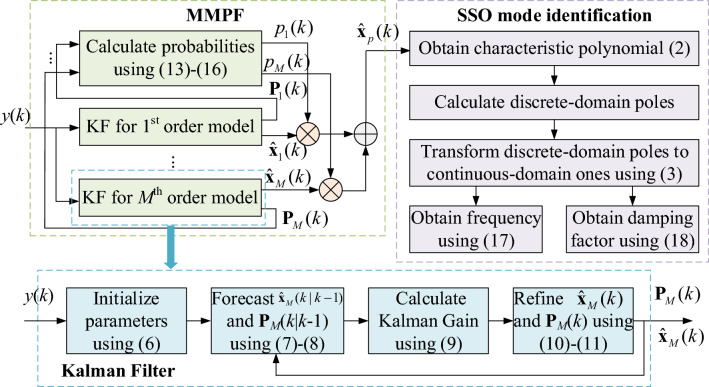
Block diagram of the MMPF-KF based SSO online monitoring method.

The block diagram of the proposed ambient data-driven SSO mode identification algorithm is shown in Fig. 2.

### Recommendations for setting the parameters

The parameters that need to be set for the MMPF-KF include the measurement noise covariance *R*(*k*), the initial value of the covariance matrix of the state vector estimation error **P**_*p*_(1), and the maximum model order *M*.

The identification accuracy of the MMPF-KF depends on the measurement noise covariance *R*(*k*). If *R*(*k*) is excessively large, the tracking results of the MMPF-KF will be imprecise. Therefore, *R*(*k*) should be chosen to be a smaller value. Note that *R*(*k*) should not be zero, while an excessively small *R*(*k*) has little impact on the tracking accuracy of MMPF-KF. Referring to the guidelines given in^25^, one effective tuning strategy is to determine the value of the parameter* R*(*k*) empirically to attain the level of estimation accuracy that is sought. In this paper, the value of *R*(*k*) in the case studies is set based on experimental observations, as the objective is not to determine the optimal configuration but rather to find the value that best satisfies the overall requirements.

The convergence of the MMPF-KF depends on the initial value **P**_*p*_(1). If the elements in **P**_*p*_(1) are too small, the MMPF-KF will fail to converge. However, if the elements in **P**_*p*_(1) are relatively larger, the convergence performance of the MMPF-KF will improve. In this paper, the values of elements in **P**_*p*_(1) are still determined based on a trial-and-error approach in order to obtain a value that satisfies the overall requirements.

The value of *M* affects the selection range of the order for the MMPF-KF. If *M* is too small and the true model order is larger than *M*, the tracking accuracy of the MMPF-KF will deteriorate. Therefore, *M* should be greater than the number of weakly damped oscillation modes present in the test system. In general, although there are multiple oscillation modes in practical power systems, the number of weakly damped modes is not infinite. Typically, there are 0–3 weakly damped modes. Therefore, setting *M* to a larger value is sufficient.

## Case studies

In order to demonstrate the performance of the proposed ambient data-driven SSO mode identification method, simulations are conducted in a classical second-order system and a test system reproducing a real SSO event in type-3 WTG-based wind farms in north China^1^. The simulation of the whole setup is carried out in SIMULINK, and results are averaged over 10 Monte Carlo trials.

### Second-order system

The mathematical model of a second-order system excited by random excitation is given by:

19 $$\ddot{y}(t) + 4\pi \xi f\dot{y}(t) + 4\pi^{2} f^{2} y(t) = F(t)$$

where *F*(*t*) is the random external driving signal. *y*(*t*) is the random response to the excitation. *ξ* and *f* are the damping ratio and natural frequency of the second-order system, respectively, and the damping ratio can be calculated by:

20 $$\xi = - \frac{\alpha }{{\sqrt {\alpha^{2} + 4\pi^{2} f^{2} } }} \times 100\%$$

where *α* is the damping factor of the second-order system.

**Figure 3:**
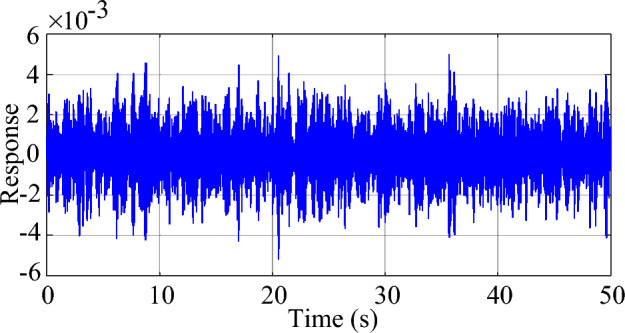
The measured response of the second-order system.

**Figure 4:**
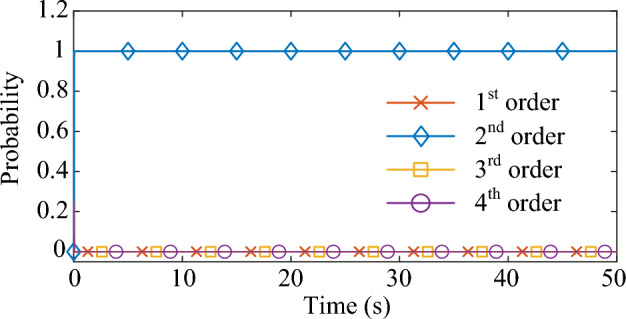
The process of the model order selection.

**Figure 5:**
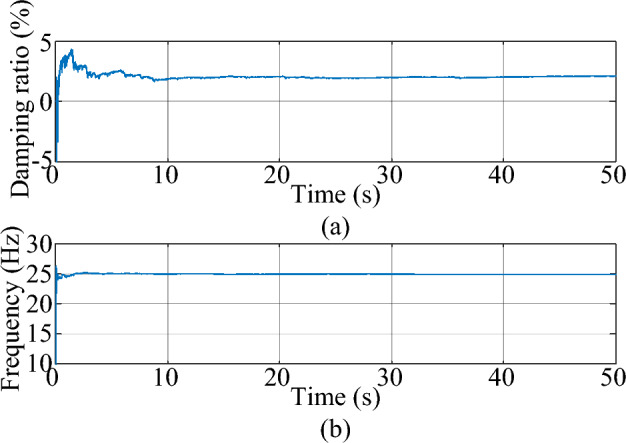
The estimated mode of the second-order system. (**a**) damping ratio (**b**) frequency.

In this case, the driving signal *F*(*t*) is a Gaussian white noise with a variance of 1. The damping ratio* x* is set at 2% and the natural frequency *f* is set at 25 Hz. Based on experimental observations, the measurement noise covariance *R*(*k*) of the MMPF-KF is set as 0.001, the initial value **P**_*p*_(1) of the MMPF-KF is set as 100*I_*p*×*p*_, and the max model order *M* of the MMPF is set as 4. The sampling rate is 200 Hz. The measured response of the second-order system is shown in Fig. 3. The process of model order selection is shown in Fig. 4. The estimated damping ratio and frequency of the system by the MMPF-KF are shown in Fig. 5.

As shown in Fig. 3, the response excited by the Gaussian white noise also exhibits noise-like properties, thus it is difficult for the ringdown data-based methods to identify SSO mode parameters from the noise-like data. As shown in Fig. 4, the MMPF selects model order 2 as the correct one immediately, which is exactly the true order of the system. As shown in Fig. 5a, the mean value of the estimated damping ratio comes to 2.02%, which is very close to the true damping ratio of the second-order system. Moreover, convergence of the estimation process occurs within 3 s. As shown in Fig. 5b, the mean value of the estimated frequency is 24.92 Hz, which is also very close to the true natural frequency of the second-order system. Meanwhile, convergence of the frequency estimation process occurs within 1 s, which takes much less time than the damping ratio estimation. From the results in this case, the MMPF-KF method can identify the mode parameters accurately and promptly from the ambient response of the second-order system, which verifies the effectiveness of the proposed method in the ideal system.

### Practical power system with type-3 WTGs

**Figure 6:**
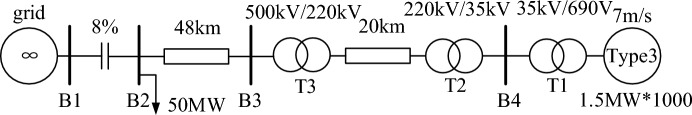
Type-3 WTG integrated power system with series compensation.

The test system employed in this case is constructed based on type-3 WTG-based wind farms in north China. This practical power system in north China has experienced multiple SSO events in the past few years, making it well-suited for the validation of analysis and monitoring methods for SSOs. The structure of the test system is shown in Fig. 6, adopting an aggregated model equivalent to the real-world system in^22^. The validity of this assumption was demonstrated in^1^ by an acceptable error analysis, which was accomplished by contrasting the outcomes of simulations with recorded data.

The parameters of the test system are shown in Tables 1 and 2.

**Table 1:** Parameters of each WTG.

Item	Value	Item	Value
Base capacity	1.5 MVA	Mutual reactance	13.68 p.u.
Stator resistance	0.0164 p.u.	Stator reactance	0.255 p.u.
Rotor resistance	0.0183 p.u.	Rotor reactance	0.222 p.u.
T1 resistance	0.01 p.u.	T1 reactance	0.06 p.u.

**Table 2:** Parameters of transmission lines and transformers.

Item	Value
Base capacity	1500 MVA
220 kV line resistance	0.02 p.u.
220 kV line reactance	0.20 p.u.
500 kV line resistance	0.01 p.u.
500 kV line reactance	0.04 p.u.
T2 reactance	0.07 p.u.
T3 reactance	0.14 p.u.

**Figure 7:**
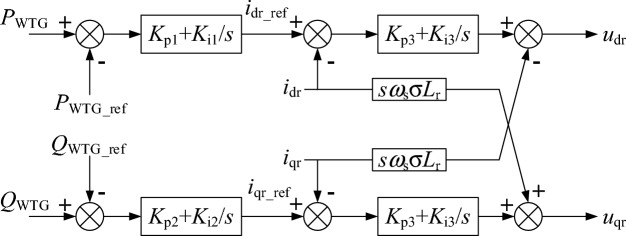
The RSC controller structure of the WTG.

**Figure 8:**
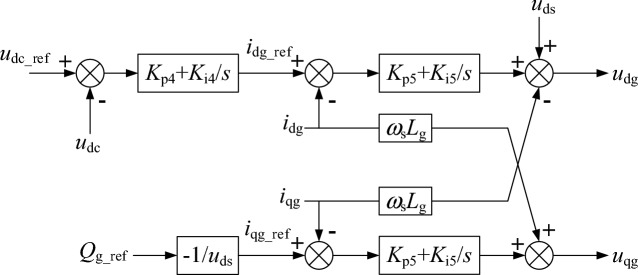
The GSC controller structure of the WTG.

The RSC controller structure of the WTG is shown in Fig. 7 and the grid side converter (GSC) controller structure of the WTG is shown in Fig. 8. The parameters of the RSC and GSC controllers are given in Table 3.

**Table 3:** Parameters of the RSC and GSC controller.

Item	Value	Description
*K*_p1_, *K*_i1_	6, 10	Proportional and integral coefficient of the RSC outer active power loop
*K*_p2_, *K*_i2_	0.4, 20	Proportional and integral coefficient of the RSC outer reactive power loop
*K*_p3_, *K*_i3_	0.23, 10	Proportional and integral coefficient of the RSC inner current loop
*K*_p4_, *K*_i4_	8, 400	Proportional and integral coefficient of the GSC outer dc voltage loop
*K*_p5_, *K*_i5_	1, 5	Proportional and integral coefficient of the GSC inner current loop

It is worth noting that the practical power system is modified by adding a constant power load of 50 MW to bus B2 in this case. All type-3 WTGs are operating in maximum power point tracking (MPPT) mode.

**Figure 9:**
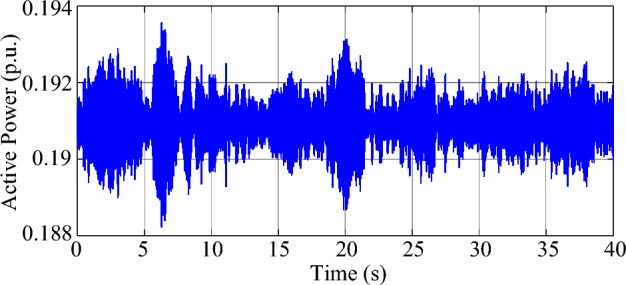
The measured active power of the type-3 WTG.

**Figure 10:**
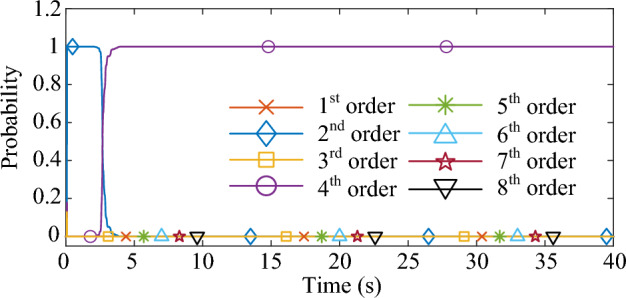
The process of model order selection.

**Figure 11:**
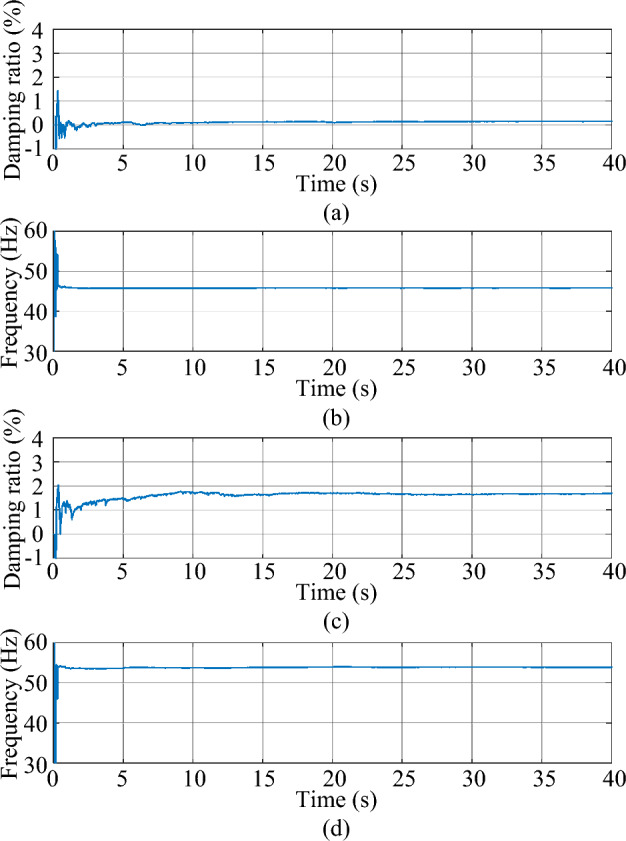
The estimated weakly damped modes of the test system. (**a**) Damping ratio of mode 1, (**b**) frequency of mode 1, (**c**) damping ratio of mode 2, (**d**) frequency of mode 2.

Under the parameters in Tables 1, 2, and 3, the test system will exhibit weakly-damped SSO modes. According to the eigenvalue analysis, there are two modes in the test system with weak damping, and the damping ratio and natural frequency of the dominant mode (mode 1) are 0.21% and 45.35 Hz, respectively. The damping ratio and natural frequency of the other weakly damped mode (mode 2) are 1.46% and 53.76 Hz, respectively. Moreover, in order to simulate the random load variation, 5% of the load at bus B2 is modeled by Gaussian noise, which has a mean value of 2.5 MW and a standard deviation of 2.5 MW, so the ambient response of the system can be excited and measured. Based on experimental observations, the measurement noise covariance *R*(*k*) of the MMPF-KF is set as 0.0001, the initial value **P**_*p*_(1) of the MMPF-KF is set as 1000*I_*p*×*p*_, and the max model order *M* of the MMPF is set as 8. The sampling frequency is 200 Hz and the dynamic simulation lasts 40 s. The measured active power of the type-3 WTG is shown in Fig. 9. The process of model order selection is shown in Fig. 10. The estimated damping ratio and frequency of the system modes by the proposed method are shown in Fig. 11.

As shown in Fig. 9, the output active power of the type-3 WTG is fluctuating as noise. As shown in Fig. 10, the MMPF selects model order 2 at first, then corrects to model order 4 after a short time and keeps it until the end of the simulation. Since there are two weakly damped modes in the test system based on the eigenvalue analysis result, selecting the model order as 4 can reflect these two modes exactly without the presence of fake modes.

As shown in Fig. 11a and c, the mean values of the estimated damping ratios in mode 1 and mode 2 are 0.18% and 1.72%, respectively, which are close to the results of the eigenvalue analysis with very fast convergence.

As shown in Fig. 11b and d, the mean values of the estimated frequencies are 45.73 Hz and 53.35 Hz, respectively, which are also very close to the results of the eigenvalue analysis. Moreover, convergences of the frequency estimation processes take much less time than damping ratio estimation. From the results in this case, the MMPF-KF method can identify the mode parameters accurately and promptly from the ambient data measured from the test system, which verified the effectiveness of the proposed method in the practical power system.

### Non-Gaussian noise scenario

**Figure 12:**
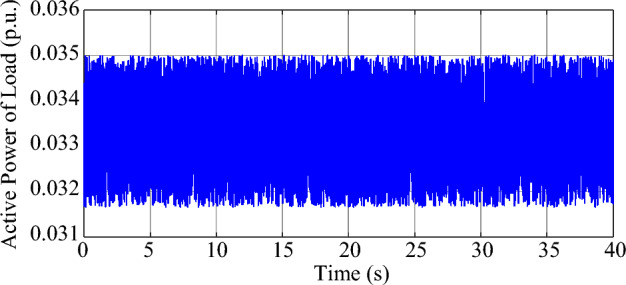
The uniformly distributed random load variation at Bus B2.

Although many studies focusing on the ambient data analysis of power systems have indicated that the stochastic load fluctuations of the system can be modeled as Gaussian white noise^28–30^, the measured noise may not follow a Gaussian distribution in some cases. In this section, the monitoring performance of the MMPF-KF will be validated in the scenario of non-Gaussian white noise. The test system employed in this case is the same as that in Section “Practical power system with type-3 WTGs”, and 5% of the load at bus B2 is modeled by the uniform white noise, which has the specified lower and upper bounds of 0 MW and 5 MW, respectively. The sampling frequency is 200 Hz and the dynamic simulation lasts 40 s. The active power of the load at bus B2 is shown in Fig. 12.

**Figure 13:**
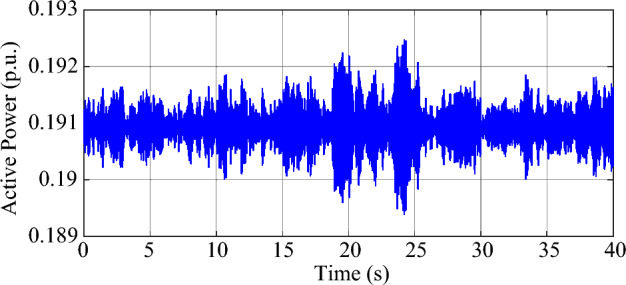
The measured active power of the type-3 WTG.

**Figure 14:**
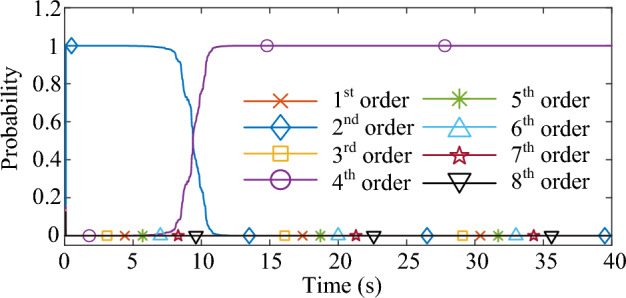
The process of model order selection.

**Figure 15:**
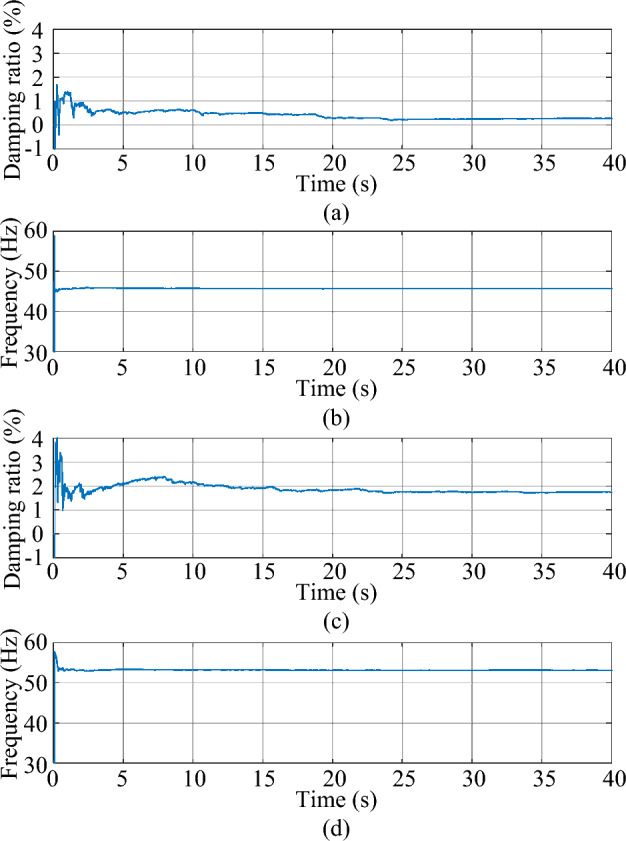
The estimated weakly damped modes of the test system. (**a**) Damping ratio of mode 1, (**b**) frequency of mode 1, (**c**) damping ratio of mode 2, (**d**) frequency of mode 2.

The measured active power of the type-3 WTG is shown in Fig. 13. The process of model order selection is shown in Fig. 14. The estimated damping ratio and frequency of the system modes by the proposed method are shown in Fig. 15.

As shown in Fig. 13, the output active power of the type-3 WTG is still fluctuating as noise. As shown in Fig. 14, the MMPF selects model order 2 at first, then corrects to model order 4 after 11 s and keeps it until the end of the simulation. Compared to the Gaussian noise scenario, the selection of the appropriate model order takes slightly more time in the non-Gaussian noise scenario. However, the results obtained remain accurate.

As shown in Fig. 15a and c, the mean values of the estimated damping ratios in mode 1 and mode 2 are 0.25% and 1.74%, respectively. As shown in Fig. 15b and d, the mean values of the estimated frequencies are 45.74 Hz and 53.19 Hz, respectively. The results obtained in this case are close to those of the Gaussian noise scenario. Besides, the convergence speed of the algorithm is also very fast in this scenario.

From the results in this case, the MMPF-KF method can also identify the mode parameters accurately and promptly from the non-Gaussian noise (e.g., uniform white noise)-excited ambient data, which further verified the effectiveness of the proposed method in the practical power system. It is noteworthy that only one particular type has been investigated in this section. Due to the various types of non-Gaussian distributed noise, further validations are required for other types of non-Gaussian distributed noise.

### Variations in wind speed

**Figure 16:**
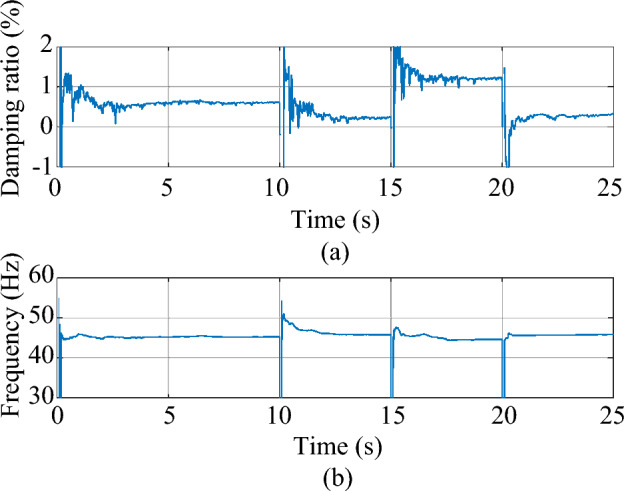
The estimated dominant mode of the test system. (**a**) Damping ratio (**b**) frequency.

In a real wind farm, the wind speed usually swings in a small-time unit. In this chapter, the monitoring performance of the MMPF-KF will be validated in these circumstances. The test system employed in this case is still based on the practical system presented in Section “Practical power system with type-3 WTGs”. The wind speed of the test system is initially 9 m/s and suddenly varies to 7 m/s at time *t* = 10 s. Then, at time *t* = 15 s, the wind speed swings to 11 m/s. Eventually, the wind speed swings back to 7 m/s at time *t* = 20 s and stays constant until the end of the simulation. The parameters of the RSC and the MMPF-KF remain the same as those in Section “Practical power system with type-3 WTGs”. The sampling frequency is 200 Hz and the dynamic simulation lasts 25 s. The estimated damping ratio and frequency of the dominant mode by the MMPF-KF are shown in Fig. 16.

As shown in Fig. 16, before *t* = 10 s, the estimated damping ratio and frequency of the test system are 0.61% and 45.37 Hz, respectively. After the sudden change of the wind speed at *t* = 10 s, the estimated damping ratio and frequency of the test system converge to 0.20% and 45.70 Hz, respectively, in a very short time. Then, when the wind speed swings to 11 m/s at *t* = 15 s, the estimated damping ratio and frequency of the test system quickly converge to 1.10% and 44.81 Hz, respectively. Eventually, when the wind speed comes back to 7 m/s *t* = 20 s, the estimated damping ratio and frequency of the test system quickly converge to 0.21% and 45.72 Hz, respectively. According to^31,32^, the damping of the type-3 WTG wind system will become weaker with a lower wind speed and stronger with a higher wind speed, while the frequency remains almost the same. The result in this case is consistent with previous research, which verifies the effectiveness of the proposed method in variable operating conditions.

### Unstable SSO scenario

**Figure 17:**
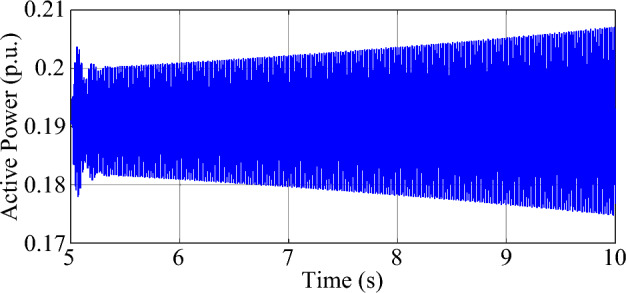
The measured active power of the type-3 WTG.

**Figure 18:**
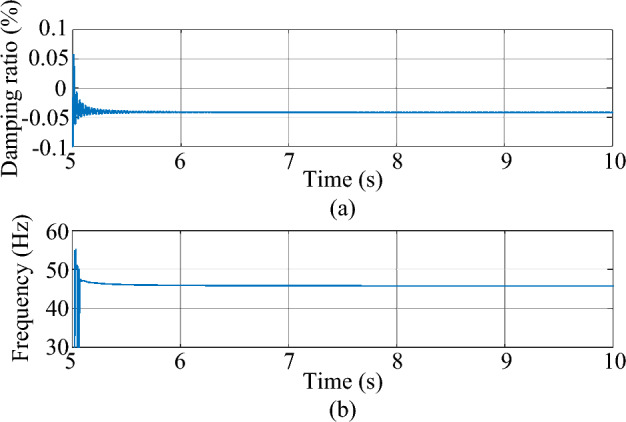
The estimated dominant mode of the test system. (**a**) Damping ratio (**b**) frequency.

This case is conducted to verify that the proposed method is still effective in identifying the unstable SSO mode based on ringdown data. The test system employed in this case is based on the one used in Case 2. The proportional coefficient of the RSC current loop in WTG is regulated as 0.3, bringing about unstable SSO in the test system. The series compensation is bypassed initially and switched in at *t* = 5 s. The parameters of the MMPF-KF and the sampling frequency remain the same as those in Case 2. The dynamic simulation lasts 10 s*.* The measured active power of WTG is shown in Fig. 17. According to Prony analysis, the damping ratio and frequency of the unstable SSO are − 0.04% and 45.68 Hz, respectively. The estimated damping ratio and frequency of the SSO by the MMPF-KF are shown in Fig. 18.

As shown in Fig. 18a and b, the mean value of the damping ratio of the unstable SSO comes to − 0.04%, and the mean value of the frequency of the unstable SSO comes to 45.80 Hz. The results are the same as those obtained by Prony analysis, showing the effectiveness of the proposed method based on ringdown data.

### Comparison with existing ambient data-based method FDD

The FDD method is a frequency domain approach that is utilized for ambient data-based SSO monitoring, as described in Ref.^15^. It is currently one of the primary methods employed for ambient data-based SSO monitoring. However, it has a major drawback in that it requires a long data window to obtain accurate results, and the computation time is also considerable. In this case, the proposed MMPF-KF method is compared to the existing ambient data-based FDD method^15^ to highlight the advantages of the proposed method.

**Figure 19:**
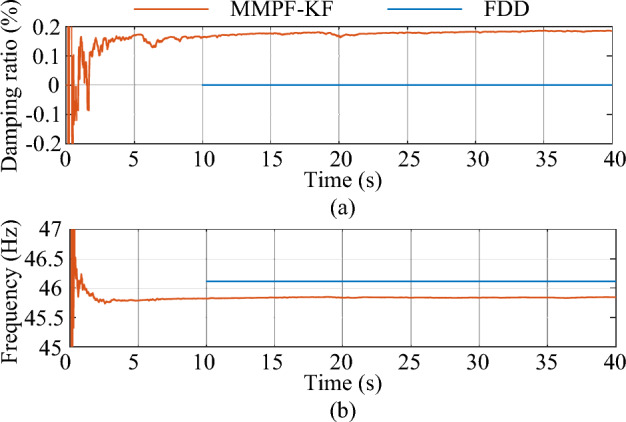
The estimated dominant mode 1 by the MMPF-KF and the FDD. (**a**) Damping ratio, (**b**) frequency.

The simulation is carried out based on Case 2, where a sliding window is employed for the FDD method to achieve continuous monitoring with a window length of 10 s. The refresh time, which indicates the duration by which the sliding window is shifted when compared to the previous window, is set to 0.005 s. The estimated damping ratio and frequency of the system mode by the proposed method and the FDD method are shown in Fig. 19.

The FDD method yielded mean values of 9.069 × 10^–4^% and 46.11 Hz for the estimated damping ratio and frequency, respectively. In comparison, the MMPF-KF method produced mean values of 0.18% and 45.73 Hz for the same parameters. The eigenvalue analysis revealed that the true damping ratio and natural frequency of the oscillation mode were 0.21% and 45.35 Hz, respectively. Thus, the proposed method exhibited higher precision. Furthermore, the analysis results in Case 2 indicated that the test system comprised two dominant weak damping modes. The proposed method accurately identified both modes, in contrast to the FDD method, which failed to identify the second weak damping mode.

Moreover, the computation time of the MMPF-KF method proposed in this study is 2.9033 s, while the computation time of the FDD method is 100.7577 s. The simulations were conducted on a workstation equipped with an Intel Core i7-7700HQ processor with 32 GB of RAM, and the computation time reported represents the average of 10 computations. Notably, the FDD method requires significantly more computation time than the MMPF-KF method, indicating that the proposed method is better suited for online monitoring applications.

### Real-time hardware experiment

**Figure 20:**
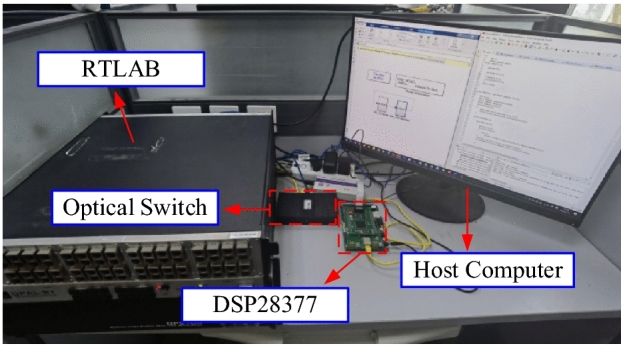
The hardware experimental platform.

To validate the effectiveness of the proposed method in practical applications, real-time hardware experiments are conducted in this section. The hardware experimental platform, as shown in Fig. 20, is constructed for this purpose. The host computer downloads the simulation model to the RTLAB, where the simulation is executed based on the test system in Case 2. The parameters of the WTG’s controller and the MMPF-KF are the same as those of Case 2. Simultaneously, the active power of the WTG is transmitted to DSP28377 through Ethernet, and DSP28377 performs digital signal processing to obtain the damping ratio and frequency of the system’s dominant SSO mode. The results are then sent back to the RTLAB as output signals, which are subsequently read by the host computer.

**Figure 21:**
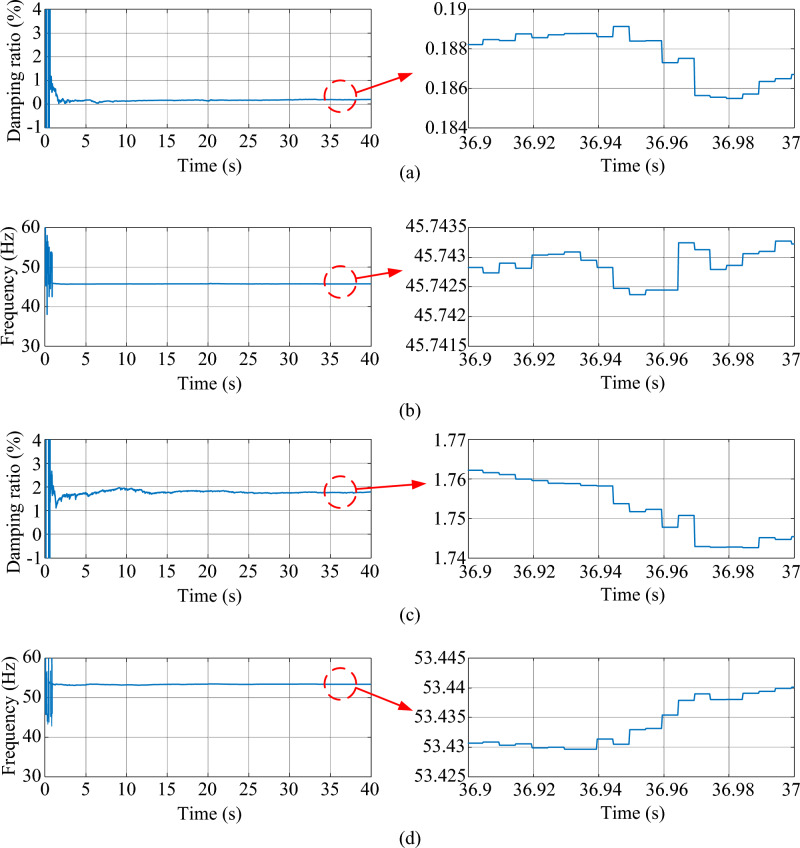
The estimated weakly damped modes of the test system in Case 2 using real-time data. (**a**) Damping ratio of mode 1, (**b**) frequency of mode 1, (**c**) damping ratio of mode 2, (**d**) frequency of mode 2.

In the real-time hardware experiment, the estimated damping ratio and frequency of the system modes by the proposed method are shown in Fig. 21.

As shown in Fig. 21, the values of the estimated damping ratios and frequencies in mode 1 and mode 2 are almost the same as those obtained by the computer simulation. From the results in this case, the MMPF-KF method can identify the mode parameters accurately and promptly from real-time ambient data, which verifies the feasibility of the proposed method in practical applications.

## Conclusions

An MMPF-KF method for SSO mode identification in type-3 WTG integrated power systems using ambient data is proposed in this paper. The KF is utilized to estimate the coefficients of the AR model to fit the measured ambient data. The damping factor and frequency in the SSO mode can be directly acquired by solving the roots of the characteristic polynomial corresponding to the AR model. Moreover, the MMPF is applied to the KF for selecting the correct AR model order. The effectiveness of the MMPF-KF method is verified in the second-order system and the practical type-3 WTG-based system. Simulation results indicate that the MMPF-KF can identify the SSO mode parameters accurately and converge in a very short time under different normal operating conditions. Moreover, the proposed method is still effective even when unstable SSO occurs. The comparison with the existing ambient data-based FDD method shows the superiority of the proposed method in both computation time and accuracy.

## Data Availability

All data generated or analyzed during this study is available from simulations of the test system in the paper. All simulations are performed on MATLAB/SIMULINK. The detailed parameters of the test system can be found in Chapter 4.2.
